# Protective Properties of the White Button Mushroom, *Agaricus bisporus*, in a Mouse Model of Colitis

**DOI:** 10.1002/mnfr.70133

**Published:** 2025-06-12

**Authors:** Elaine Dempsey, Aaron M. Walsh, Supriya Yadav, Jude Wilson, Frederick J. Sheedy, Sinead C. Corr

**Affiliations:** ^1^ School of Genetics & Microbiology Trinity College Dublin Dublin Ireland; ^2^ School of Biochemistry & Immunology Trinity College Dublin Dublin Ireland; ^3^ MBio, Monaghan Mushrooms Tyholland, Co. Monaghan Ireland; ^4^ School of Microbiology and APC Microbiome Ireland University College Cork Cork Ireland

**Keywords:** colitis, inflammation, microbiome, mushroom, oxidative stress

## Abstract

Previous work demonstrated the promising immunomodulatory potential of a naturally derived whole mushroom powder (WMP) from the white button mushroom, *Agaricus bisporus*. Here, we further investigate the protective properties of WMP in a mouse model of colitis. An in vitro digested WMP (IVD‐WMP) reduced permeability of intestinal epithelial Caco‐2 and HT‐29‐MTX cell monolayers to FITC dextran. In vivo, WMP orally administered to mice as a pretreatment before induction of dextran sulfate sodium (DSS)‐induced colitis. Though statistically significant decreases in disease scores were not reported, we observed an antiinflammatory and antioxidative stress profile in the colon. Additionally, 16S ribosomal RNA (16S rRNA) microbiome analysis revealed differences in bacterial abundance associated with WMP pretreatment, including a decrease in *Allobaculum* species associated with inflammatory bowel disease. In a DSS‐colitis recovery model, WMP promoted recovery as evidenced by improved weight gain, reduced stool scores, reduced IL‐1β levels, and myeloperoxidase (MPO) activity in colonic tissue. This work demonstrates the health benefits associated with the consumption of the white button mushroom, including support of intestinal barrier integrity combined with antioxidant and antiinflammatory activity.

## Graphical abstract

In this study, whole mushroom powder (WMP) from white button mushrooms was tested for gut health benefits. In vitro digested WMP (IVD‐WMP) could reduce the permeability of a gastrointestinal cell layer. In mouse studies, WMP was tested as a preventative and a treatment for dextran sulfate sodium (DSS) induced colitis. WMP could improve recovery, reduce inflammation and oxidative stress, and promoted healthier gut bacteria. This suggests WMP could help support digestive wellness and recovery from gut inflammation.

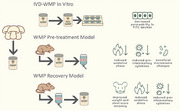

**Abbreviations**
16S rRNA
: 16S ribosomal RNA
ANOVA: analysis of variance
DAI: disease activity index
DSS: dextran sulfate sodium
EGFR: epithelial growth factor receptor
H&E: hematoxylin and eosin
IBD: inflammatory bowel disease
IVD‐WMP: in vitro digested whole mushroom powder
MDA: malondialdehyde
MPO: myeloperoxidase
MUC: mucin
ROS: reactive oxygen species
SOD: superoxide dismutase
TNF: tumor necrosis factor
WMP: whole mushroom powder
ZO: zonula occludens

## 1 Introduction

Mushrooms have been celebrated for millennia for their culinary, nutritional, and medicinal properties. The ancient Egyptians, Romans, and Mayans revered mushrooms as “immortality food,” “food of the Gods,” and “flesh of the Gods,” respectively [1, 2], while traditional healing practices worldwide utilized various mushroom species for their therapeutic effects and spiritual healing properties [3, 4]. This legacy continues into modern scientific research, revealing a range of bioactive properties of mushrooms including immunomodulatory, antidepressant, antitumor, antidiabetic, antimicrobial, and antioxidative activities [5, 6, 7, 8, 9, 10, 11]. Dietary consumption of mushrooms has been associated with a reduced risk of various diseases, such as obesity, diabetes, cancer, and digestive conditions [12, 13, 14, 15]. The health benefits are believed to be driven by mechanisms such as direct or indirect interactions with the immune and nervous systems, neutralizing free radicals, and regulation of the gut microbiota [15, 16, 17, 18, 19].

Despite representing one of the most widely consumed mushroom species worldwide, the health‐promoting effects of the white button mushroom (*Agaricus bisporus*) remain underexplored. Some studies suggest potential benefits for vitamin D levels, metabolic syndrome, immune function, gastrointestinal health, and cancer, although further research is necessary to confirm these effects [20]. Modern cultivation techniques allow the production of *A. bisporus* mushrooms that are enriched for various vitamins and nutrients, including vitamin B12, B6, selenium, and most commonly vitamin D, augmenting their potential for immune support [21, 22]. Indeed, we have recently reported on the significant antioxidant properties of normal and enriched whole mushroom powders (WMPs) derived from *A. bisporus* in terms of in vitro free radical scavenging activity, and total polyphenol and ergothioneine content [22]. In this study, WMP from *A. bisporus* was shown to be a significant source of antioxidants compared to a selection of commercially available mushroom powders, including Shitake, Chaga, Reishi, and Lion's Mane. Compared to commercially available mushroom powders, nutritionally enriched WMP had significantly higher 2,2‐diphenyl‐1‐picrylhydrazyl (DPPH) scavenging activity, nitric oxide (NO) radical scavenging activity, hydroxyl radical scavenging activity, higher levels of ergothioneine and polyphenol content, as well as lower IC_50_ values, indicative of better antioxidant capacity. Additionally, we have shown that these powders regulate innate and trained immunity in mouse and human macrophages, demonstrating a dampening of innate immune response, an ability to promote trained immunity, as well as a protection in mouse models of sepsis and colitis [23, 24]. Previously, extracts from *A. bisporus* have resulted in decreased expression of cyclooxygenase‐2 (COX‐2) and prostaglandin F2α ‐receptor (PF2AR) in LPS‐ and/or tumor necrosis factor (TNF)‐α‐activated Caco‐2 cells, with increased expression of nuclear factor (erythroid‐derived 2)‐like 2 (NRF‐2) [25]. Kohno et al. [26] isolated the compound 2‐amino‐3H‐phenoxazin‐3‐one (APO) from *A. bisporus* mushroom extracts and demonstrated its potent antiinflammatory and immunoregulatory properties. APO was shown to inhibit NO, prostaglandin‐E_2_ (PGE_2_), IL‐6, and IL‐12 in LPS and/or IFNγ‐stimulated macrophages, while also inducing antigen‐stimulated Th cells to produce the antiinflammatory cytokine IL‐4. Methanolic extracts from *A. bisporus* achieved similar reductions in NO production and expression of iNOS, IL‐1β, and IL‐6 in LPS‐treated macrophages [27]. Notably, in a recent study of purified polysaccharide fractions from *A. bisporus*, microbiota‐dependent beneficial effects were obtained in a murine colitis model [28]. Additionally, a recent study on the fungal microbiome in ulcerative colitis patients found decreased *Agaricus* species in the gut during endoscopic activity versus during endoscopic remission [29].

Inflammatory bowel disease (IBD), encompassing Crohn's disease and ulcerative colitis, is a chronic inflammatory condition of the gastrointestinal tract characterized by a dysregulated immune response, mucosal barrier disruption, and gut microbiota dysbiosis [30]. A westernized diet is believed to be a key risk factor in the development of IBD [31]. IBD presents a significant and growing global health burden, which greatly impacts patients’ quality of life [32]. Current treatment strategies, which primarily focus on symptom management, include corticosteroids, immunosuppressants, and biologics, all of which are associated with potential serious adverse effects with long‐term use [33, 34, 35]. Therefore, the exploration of alternative and complementary therapies, particularly those derived from natural sources, remains a valuable research area.

This study further explores the potential of *A. bisporus* WMP to protect the intestinal barrier, improve symptoms, and mitigate inflammation in the context of colitis. To do this, we produced a simulated in vitro digested WMP (IVD‐WMP) product and tested its effects on two human gastrointestinal epithelial cell lines (Caco‐2 and HT‐29‐MTX). We then evaluated *A. bisporus* WMP in both pretreatment and recovery models of DSS‐induced colitis in mice. The goal of this research is to confirm the beneficial effects of *A. bisporus* on the gastrointestinal barrier, elucidate the underlying mechanisms, and contribute to the development of novel dietary or therapeutic strategies for IBD management.

## 2 Experimental Section

### 2.1 Mushroom Powder

Mushroom powder used in this study was from *A. bisporus* mushroom crops obtained from MBio Ltd., part of the Monaghan Mushrooms group, as previously described [36]. Simulated in vitro digestion of WMP was carried out for use in cell culture experiments as described previously [23]. WMP or IVD‐WMP was prepared fresh by resuspending in PBS or the appropriate culture media, sonicating in a water bath for 10 min, incubating overnight, and rolling at 4°C to dissolve. Solutions for use in vitro were additionally passed through 0.2 µM filters (15206869, Fisher) before use to sterilize.

### 2.2 Dosage Information

Dosage was determined as described previously [23]. In vitro, a standard dose of 1 mg/mL IVD‐WMP was used. For in vivo experiments, an oral bolus WMP dose equivalent to 500 mg/kg body weight was delivered via oral gavage, resuspended in PBS to a maximum volume of 100 µL. This equates to 10 mg WMP in a 20 g adult mouse. The equivalent human dose is 40 g in an 80 kg adult. DSS control mice received PBS only, and healthy control mice were subject to a sham gavage where the gavage needle was inserted but no liquid dispensed. All gavage procedures were carried out at the same time in the morning. In the pretreatment trial, gavage was performed every second day for 14 days due to restrictions in performing this procedure daily for an extended period. In the recovery trial, gavage was performed daily for 5 days.

### 2.3 Cell Culture

Caco‐2 or HT‐29‐MTX cells were maintained at 37°C with 5% CO_2_ and 95% relative humidity between Passages 65–75 for caco‐2 and Passages 25–30 for HT‐29‐MTX. Cells were cultured in growth medium (GlutaMAX DMEM 11971025, 10% FBS 16030074, 1% Penicillin‐Streptomycin 15640055, Thermo Fisher) with the addition of nonessential amino acids (M7145, Sigma) for HT‐29‐MTX cells only. For use in experiments, cells were seeded at a density of 3 × 10^4^ cells per transwell filter (0.4 µm pore size, Costar, Corning). Cells were allowed to differentiate into a cell monolayer for 21 days before treatment. Cells were treated with 1 mg/mL IVD‐WMP and incubated at 37°C for 24 h.

### 2.4 In Vitro Permeability Assay

Integrity of the cell monolayer was assayed by measuring permeability to FITC dextran as previously described [37]. Briefly, following the treatment incubation period, media in the apical compartment of the transwell was replaced with 1 mg/mL FITC dextran 4 kDa (FD4; 46944, Sigma–Aldrich) in serum‐free media. Aliquots from the basal compartment of the transwell were taken 4 h later. Fluorescence in these samples was measured at *λ*_EX_ = 485 nm and *λ*_EM_ = 535 nm to determine FD4 concentration in the basal compartment.

### 2.5 In Vivo Studies

C57BL/6 mice were maintained in ventilated cages at 21 ± 1°C, humidity 50% ± 10%, and with a 12 h‐light/12 h‐dark cycle under specific pathogen‐free conditions according to Irish and European Union regulations. Food and water were monitored and available ad libitum throughout experiments unless otherwise stated. All animal experiments were carried out according to the recommendations of Trinity College Dublin's Animal Research Ethics Committee and the Irish Health Products Regulatory Authority, the competent authority responsible for the implementation of Directive 2010/63/EU on the protection of animals used for scientific purposes in accordance with the requirements of the S.I No 543 of 2012.

### 2.6 DSS‐Induced Colitis

The DSS‐induced colitis model is widely used as a robust model to study acute gastrointestinal inflammation similar to ulcerative colitis [38]. Colitis was induced by administration of DSS (36–50 kDa, MP Biomedicals) ad libitum via drinking water as previously described [39]. Control mice received normal drinking water throughout (Healthy). Once DSS administration had been initiated, mice were scored at the same time daily for body weight loss (as a % of weight lost compared to weight on Day 0 of DSS), stool consistency, and fecal blood using a 0–4 scoring system for each parameter as outlined in Table 1. The disease activity index (DAI) was calculated daily for each mouse based upon the sum of individual scores of those three parameters. Fecal occult blood was measured using the Hema‐Screen testing kit (Immunostics). At the end of the trial, mice were sacrificed by CO_2_ asphyxiation. Colons were removed and colon length was measured as an indication of colonic inflammation. Proximal and distal colon sections were taken and either fixed in 10% formalin for histology or snap frozen at −70°C until further analysis.

**TABLE 1:** Scoring criteria for DAI.

Score	Weight loss (%)	Stool consistency	Blood
0	<1	Solid	None
1	1–3	Soft, well‐formed pellet	Faint positive test
2	4–6	Softer, looser pellet	Clear positive test
3	7–9	No form, diarrhea	Visible red stool
4	>10	Liquid diarrhea	Visible blood

### 2.7 In Vivo Trial Schedules

A pretreatment and a recovery trial were performed to determine the impact of WMP on the development of colitis and on recovery from established colitis, respectively. The experimental timeline for the pretreatment trial is illustrated in Figure 2A. For the pretreatment trial, mice were given WMP (500 mg/kg) via oral gavage every second day for 9 days before DSS administration and also every second day during DSS administration (14 day period in total) (Figure 2A). Dextran sulfate sodium (DSS; 2.5% w/v) was given for the final 5 days of those 14. All mice were given normal drinking water for two further days before cull. Mice were culled 2 days following cessation of DSS, as this is typically the timepoint at which physiological symptoms are most severe. The experimental timeline for the recovery trial is illustrated in Figure 2E. For the recovery trial, mice were given DSS (2% w/v) for 4 days before commencement of WMP oral gavage (500 mg/kg), which was administered daily for 5 days (Figure 2E). WMP groups were compared to mice receiving DSS and PBS vehicle only (indicated DSS).

### 2.8 Mucosa‐Blood Flux FITC‐Dextran

Intestinal permeability in the pretreatment trial was assayed by translocation of FITC dextran 40 kDa (FD40; FD40S, Sigma–Aldrich). At 4 h before cull, mice received 80 mg/kg FD40 in 150 µL sterile PBS via oral gavage. Food was removed from the cage. Mice were culled by CO_2_ asphyxiation, blood was collected immediately and left to stand at room temperature for at least 30 min. Serum was collected by centrifugation at 4°C for 12 min at 1000 × *g*. Fluorescence of FD40 is measured in the serum at *λ*_EX_ = 485 nm and *λ*_EM_ = 535 nm.

### 2.9 Histology

Sections from the distal colon of each mouse were obtained at the time of cull and subsequently fixed in 10% buffered formalin, embedded in paraffin wax, sliced on a microtome at 5 µm thickness, and placed on polysine adhesive slides. Slides were baked at 55°C for 15 min. Hematoxylin and eosin (H&E) staining was performed to assess crypt length, tissue damage, and neutrophil infiltration. Alcian blue staining was performed to assess goblet cell loss. The staining steps were performed by a Leica Autostainer XL (Leica Microsystems). For H&E: xylene (x2), absolute alcohol (x2), tap water, hematoxylin (4 min), tap water, acid alcohol, tap water, eosin (2 min), tap water, absolute alcohol (x2), and xylene (x2). For Alcian blue: xylene (x2), absolute alcohol (x2), tap water, Alcian blue (pH 1; 5 min), tap water, nuclear fast red (5 min), tap water, absolute alcohol (x2), and xylene (x2). Tissue slices were imaged using an Olympus BX51 upright microscope. All slices were blind scored. For H&E sections, tissue damage and neutrophil infiltration were scored according to the scoring system as outlined in Table 2. Crypt length was measured on 10 separate crypts/areas per slice. Goblet cell loss was measured using QuPath (RRID: SCR_018257) [40] by isolating the epithelial layer, thresholding to the goblet cell stain, and measuring the area of stain as % of epithelial area.

**TABLE 2:** Scoring criteria for H&E.

	Inflammation severity	Inflammation extent	Crypt damage	Percent involvement
0	None <10%	Occasional cells in LP	None	None
1	Mild 10–25%	Mucosa	Basal 33%	Up to 25%
2	Moderate 26%–50%	Mucosa and submucosa	Basal 66%	26%–50%
3	Severe 51%+	Transmural	Crypt loss; surface epithelium present	51%–75%

### 2.10 16S Sequencing and Analysis

To determine the effects of WMP on the gut microbiota composition, 16S ribosomal RNA (16S rRNA) sequence analysis was performed on fecal samples from each trial. For the pretreatment trial, fecal samples were taken on Day 0 (start WMP), on Day 9 of WMP administration (start DSS), and on Day 16 (cull). For the recovery trial, samples were taken on Day 0 (start DSS), on Day 4 (end DSS, start WMP), and on Day 9 (cull). DNA extraction was performed using a Power‐fecal‐pro DNA extraction kit (51804, Qiagen) according to the manufacturer's protocol. DNA samples were quantified using a Qubit Broad Range DNA kit (Thermo Fisher) and diluted to 5 ng/µL. 16S metagenomics libraries were prepared using a miniaturization protocol based on the 16S metagenomic library preparation guide (Illumina). In brief, an Echo 525 (Beckman Coulter) was used to combine 0.5 µL DNA, 2.5 µL Kapa Hifi HotStart Ready Mix, and 1 µL of each of the primers (at 1 µM) in individual wells of a 96‐well plate. The plate was spun briefly, and amplification was performed according to the 16S metagenomic library preparation guide (Illumina) with 30 cycles in place of 25. Following amplification, the volume of each sample was increased to 20 µL using molecular grade water, and the samples were cleaned using a 0.8 X ratio of Ampure beads (Beckman Coulter) on a Beckman i7 (Beckman Coulter). The index PCR was again set up using the Echo 525 to dispense the reagents as follows: 1 µL cleaned PCR product, 2 µL Unique Duel Indices (IDT), 5 µL Kapa Hifi HotStart Ready Mix, and 2 µL molecular grade water. Plates were spun briefly, and PCR was performed as described in the 16S metagenomic library preparation guide (Illumina). Samples were then quantified using a Qubit High Sensitivity kit (Thermo Fisher) and pooled at equal concentration. The final pool was further cleaned using a 0.7 X ratio of Ampure beads (Beckman Coulter) and quantified using the Qubit High Sensitivity kit (Thermo Fisher). It was then sequenced on an Illumina NextSeq 2000 using NextSeq1000/2000 P1 Reagents (600 cycles) according to the manufacturer's guidelines (Illumina). Sequencing data was processed using QIIME 2 (version 2024.5.0) (RRID:SCR_021258) [41]. Primer sequences were removed from the demultiplexed reads using the command “qiime cutadapt trimmed‐paired”. Quality filtering, paired‐end read merging, chimera removal, and amplicon sequence variant (ASV) clustering were performed using the command “qiime dada2 denoise‐paired”. Taxonomic assignment of ASVs was performed using the Greengenes 13.8 (RRID:SCR_002830) [42] with the command “qiime feature‐classifier classify‐sklearn”. Subsequently, downstream analysis was performed in R. Alpha diversity was measured using the Shannon index, which was computed using the diversity function from vegan (version 2.4.6) (RRID:SCR_011950). PERMANOVA was performed using the adonis2 function from vegan (version 2.4.6). Principal coordinate analysis (PCoA) was performed using the pcoa function from ape (version 5.7.1) (RRID:SCR_017343). The Wilcoxon signed‐rank test was used to test the significance for differential abundance analysis, and the resulting *p* values were corrected using the Benjamini–Hochberg method. Data was visualized using ggplot2 (version 3.5.1) (RRID:SCR_014601).

### 2.11 Cytokine Quantification

Cytokine concentration was determined using Invitrogen Uncoated ELISA kits (Thermo‐Fisher) for mouse TNF (88‐7324), IL‐1𝛽 (88‐7013), IL‐6 (88‐7064), and IL‐10 (88‐7105) as per the manufacturer's protocol.

### 2.12 Oxidative Stress Assays

Myeloperoxidase (MPO) activity in colon tissue was assessed using a slightly modified version of a previously described assay [43]. The modifications are as follows: 3,3′,5,5′‐tetramethylbenzidine (TMB; 15808028, Fisher Scientific) was used a substrate for the assay, and MPO activity was expressed in arbitrary units (AU). Other oxidative stress markers were measured in colon tissue using specific assay kits (Sigma) for superoxide dismutase (SOD) activity (CS0009; Sigma), and lipid peroxidation (malondialdehyde [MDA]) (MAK085; Sigma) according to manufacturer's protocols.

### 2.13 RT‐qPCR

RNA was isolated using the PureLink RNA mini kit (12183018A, Invitrogen) according to manufacturer's protocol, quantified using a Nanodrop 2000 UV‐visible spectrophotometer, and DNase‐treated according to manufacturer's protocol (EN0521, Thermo Scientific). Total RNA was reverse transcribed with a high‐capacity cDNA reverse transcription kit (4368814, Applied Biosystems), and cDNA was amplified using SYBR green‐based real‐time PCR (A25742, Applied Biosystems). SYBR RT‐qPCR results were analyzed by the CT method [2‐(ΔΔCT)] to compare genes of interest to endogenous control genes. Primer sequences are listed in Table 3.

**TABLE 3:** Primer sequences for RT‐qPCR.

		Forward primer	Reverse primer
β‐Defensin	Human	TCTGAGATGGCCTCAGGTGGT	GTAAAGATCGGGGACGCAGAA
Claudin1	Mouse	ATGACCCCTTGACCCCCATC	GGAGCAGGAAAGTAGGCACC
Claudin1	Human	GACCCTATGACCCCCAGTCAATG	TTTTCGGGGACAGGAACAGC
Claudin2	Mouse	CCTGGGATTGTGCTTGAGGT	TGACCCCCATCACCCACAGA
Cadherin	Mouse	CCCAGAGACTGTGTGCATTT	TGGCAATGGGTGAACCA TCA
e‐Cadherin	Human	CTGGTGGTCAACGCTCCTGAC	CACCTGACCCTGTGTACTGGGT
EGFR	Mouse	TCTTCAAGGAGTTGAAGTGTG	TGTACGCTTTCGAACAATGT
EGFR	Human	AGCGCTACCTTCTTCATTCAG	CACGTGCTCCATGTCTTCTTT
GAPDH	Mouse	TGTGTCCGTGGTGTGA TGGA	CCTGCTTCACCACCTTCTTGAT
GAPDH	Human	TCGACAGTCAGCCGCATCTT	GCCCAATACGACCAAATCCGT
MUC1	Mouse	AAGCGTAGCCCTCATGAGGA	GGGGTGACTTGCTCCTACAA
Occludin	Mouse	TCCGTAGGGCCTTTTGAA	GGTGCATATGATTGGGTTTG
Occludin	Human	TCAGGGAATACCCACTACATT CAG	CATCAGCAGCCACGTTACTCTTCAC
ZO1	Human	CCTGAGTTTGACAGTGGA GT	GCTGAAGGACTACAGGAATAG
ZO2	Mouse	GCACCCTGACATCTATGCG	CACTGCCGTAGCTTCCTCTG

### 2.14 Statistical Analysis

Data were analyzed and graphs were generated using GraphPad Prism v8.0 (GraphPad Software; RRID:SCR_002798), except for the microbiome data analysis described Section 2.10. Data were presented as mean ± SEM. Data were analyzed by *t* test, one‐way analysis of variance (ANOVA), or two‐way repeated measures ANOVA as appropriate. If any statistically significant differences were revealed by ANOVA, post hoc comparisons were performed using a Tukey test. In all cases, a *p* value of < 0.05 was considered statistically significant.

## 3 Results

### 3.1 *A. bisporus* IVD‐WMP Reduces Epithelial Barrier Permeability In Vitro

**FIGURE 1:**
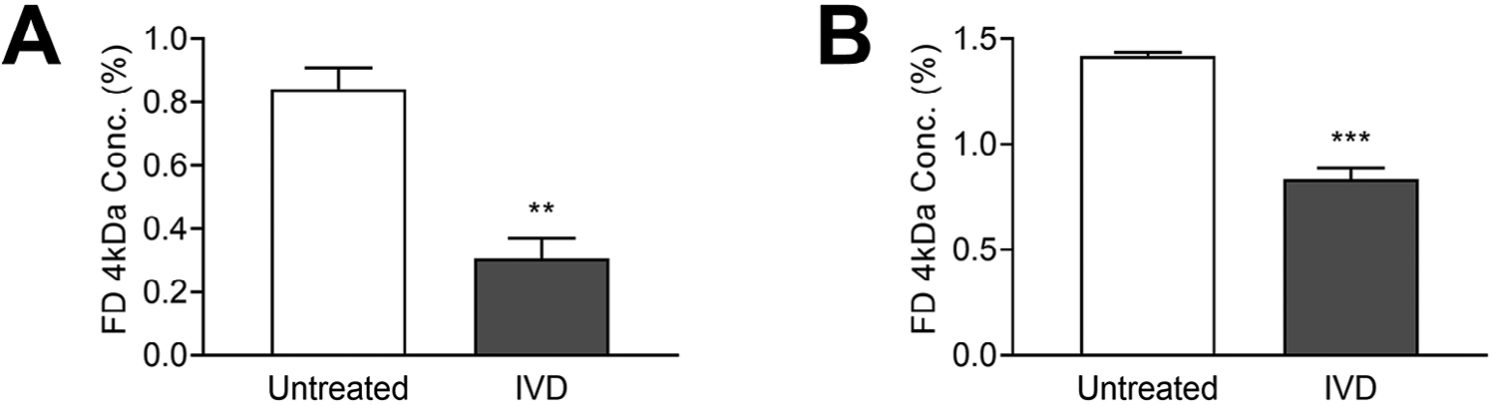
*A. bisporus* IVD‐WMP reduces epithelial barrier permeability in vitro. Caco‐2 and HT‐29‐MTX cell lines were grown on transwell plates and pretreated for 24 h with *A. bisporus* IVD‐WMP (1 mg/mL) before washing and addition of fresh media containing FITC dextran (4 kDa; 1 mg/mL). FITC concentration was measured in the basal compartment 4 h later for Caco‐2 (A) and HT‐29‐MTX (B) cells. Data are mean ± SEM of *n* = 3 independent experiments. **p value < 0.01, ***p value < 0.001 determined using unpaired *t* test. IVD‐WMP, in vitro digested whole mushroom powder.

To mimic digestion and to assess the impact of exposure of the gut epithelial barrier to digested WMP, an in‐vitro digestate (IVD) of WMP (IVD‐WMP) was prepared. Pretreatment of Caco‐2 cell monolayers with IVD‐WMP significantly reduced the translocation of FITC‐dextran into the basal compartment of the transwell system (Figure 1A). A similar effect was observed in HT‐29‐MTX cells, where IVD‐WMP pretreatment significantly reduced FITC translocation at 4 h (Figure 1B). Though RT‐qPCR analysis of barrier‐related gene transcription in these cells indicated no significant changes, there was a slight trend for increased transcription of β‐defensin1 and epithelial growth factor receptor (EGFR) in Caco‐2 cells (Figure S1A, D). These data suggest that IVD‐WMP may enhance the integrity of gastrointestinal epithelial cell layers. We next sought to investigate this further in vivo.

### 3.2 *A. bisporus* WMP Pretreatment and DSS‐Induced Colitis in Mice

**FIGURE 2:**
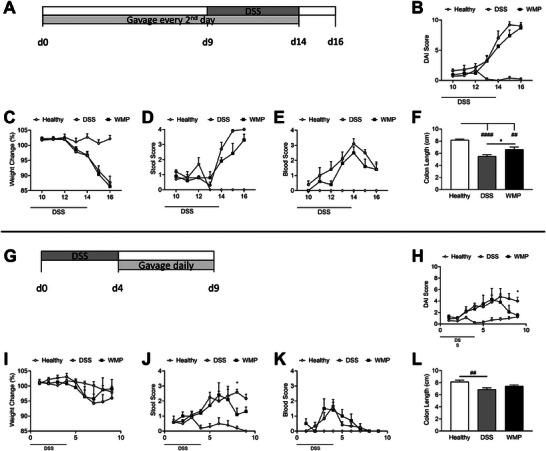
WMP gavage can reduce symptoms of DSS‐induced colitis in recovery. *A. bisporus* WMP (500 mg/kg) or vehicle control (PBS) was delivered by oral gavage to C57BL/6 mice before, or following, administration of DSS according to the trial schedule for a pretreatment (A–F) or recovery (G–L) model. Once DSS was initiated, mice were scored daily for symptoms of colitis alongside a healthy control group (sham gavage and no DSS). Disease activity index (DAI) was plotted (B, H) as the sum of the scores for weight loss (C, I), stool consistency (D, J), and fecal blood score (E, K). Colon length was measured at cull (F, L). Data are mean ± SEM of *n* = 5–6 mice per group. *p value < 0.05, **p value < 0.01, ****p value < 0.0001 determined using two‐way repeated measures ANOVA or one‐way ANOVA with Tukey post‐hoc multiple comparisons where appropriate. (# Relative to healthy control; * WMP relative to DSS control). ANOVA, analysis of variance; DSS, dextran sulfate sodium; WMP, whole mushroom powder.

Gastrointestinal inflammation and intestinal barrier dysfunction were modeled in mice by the administration of DSS via drinking water. Development of colitis was characterized by body weight loss, diarrhea, and bloody stools. In the pretreatment trial, *A. bisporus* WMP was administered orally by gavage before and during DSS administration to assess its impact on susceptibility to development of the disease (Figure 2A). Although not statistically significant, the combined DAI score (Figure 2B) was consistently lower in the WMP group compared to DSS‐only mice every day bar one (Figure 2B). There was no difference in weight loss between WMP and DSS‐only mice (Figure 2C). Although not statistically significant, stool consistency score indicative of diarrhea (Figure 2D) and blood score (Figure 2E) appeared lower on most days in the WMP group compared to DSS‐only mice. Colon length was significantly reduced in the DSS group compared to the WMP pretreated group (Figure 2F), indicating a significant protective effect of WMP, as colon shortening is typically associated with inflammation in this model.

### 3.3 *A. bisporus* WMP Improves Recovery From DSS‐Induced Colitis in Mice

Next, we assessed the impact of WMP on recovery from established colitis (Figure 2G–L). In this model, DSS‐colitis was induced first followed by administration of WMP or vehicle control by gavage during a recovery period (Figure 2G). WMP‐treated mice showed significant improvement in overall DAI score on Day 9 (Figure 2H). No statistically significant effects of WMP were observed for weight loss, although weight loss appeared lowed in WMP‐treated mice toward the end of the recovery period (Figure 2I). WMP‐treated mice showed significant improvement in stool consistency, indicative of reduced diarrhea, on Day 8 (Figure 2J) compared to DSS‐only mice. No statistically significant effects of WMP were observed for blood scores (Figure 2K). Significant colon shortening observed in DSS‐only mice compared to healthy controls was not observed in WMP‐treated mice compared to healthy controls suggesting improved recovery following WMP administration (Figure 2L). Taken together, these results demonstrate that WMP administration enhances recovery from established colitis.

### 3.4 Pretreatment With *A. bisporus* WMP Supports Barrier Integrity Against DSS‐Induced Colitis

**FIGURE 3:**
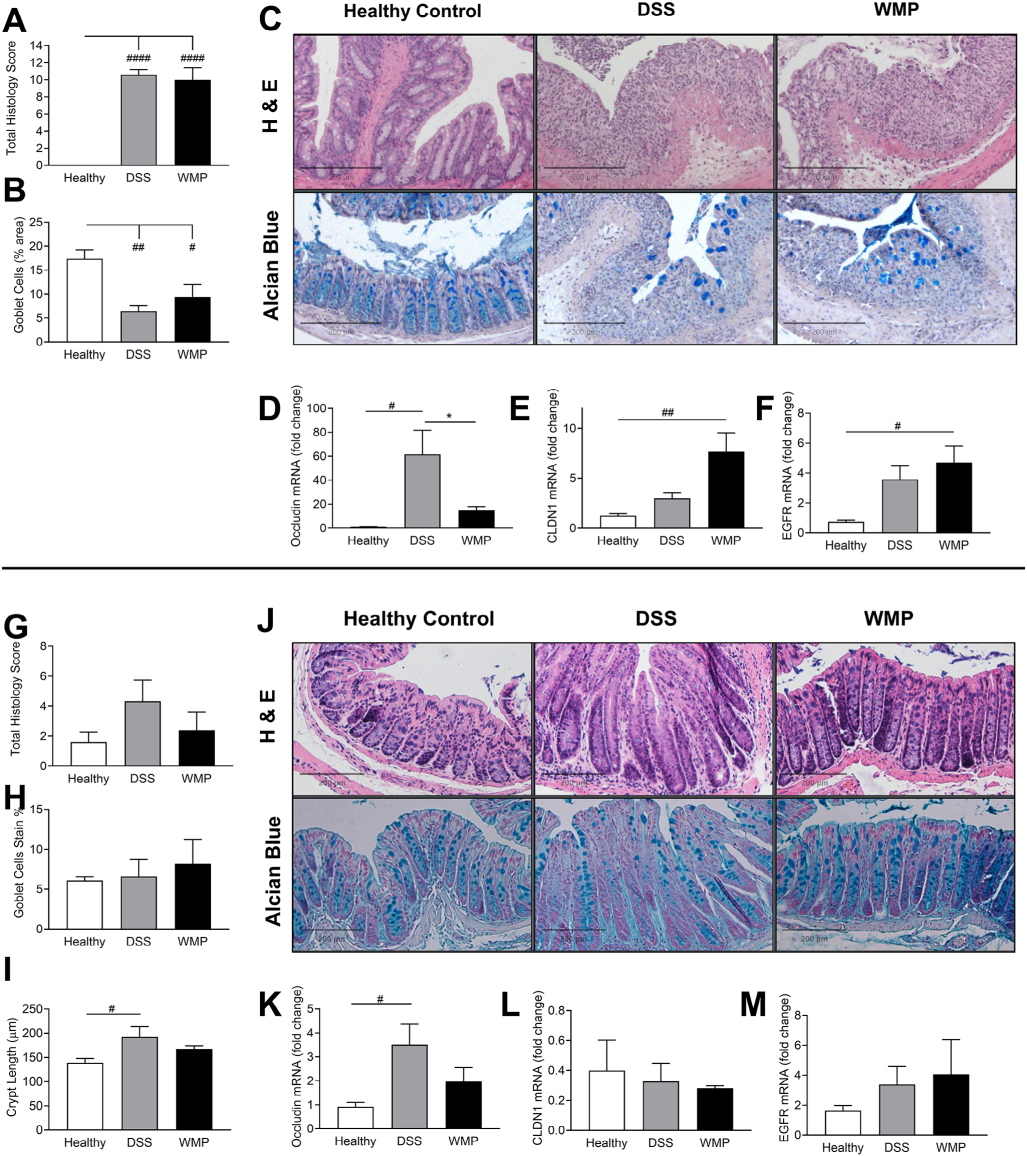
WMP has little effect on barrier integrity in DSS‐induced colitis. Distal colon tissue from pretreatment (A–F) and recovery (G–M) trials were fixed in formalin (10%), and 5 µm slices were histological analysis performed by the H&E method and the Alcian blue method. Representative images for both are shown (C, J; 100X magnification; scale bar = 200 µm). H&E images were scored for tissue damage and neutrophil infiltration to give a combined histology score (A, G). Alcian blue images were assessed for positive goblet cell stain (B, H). Additionally, in the recovery trial crypt length could be measured (I). RNA was extracted from distal colon tissue, and fold change in expression of genes related to barrier integrity was measured: occludin (D, K), claudin1 (E, L), EGFR (F, M). Data are mean ± SEM of *n* = 3–5 mice per group. *p value < 0.05, **p value < 0.01, ****p value < 0.0001, determined using one‐way ANOVA with Tukey post‐hoc multiple comparisons where appropriate. (# Relative to healthy control; * relative to DSS control). ANOVA, analysis of variance; DSS, dextran sulfate sodium; H&E, hematoxylin and eosin; WMP, whole mushroom powder.

Histological analysis of colonic tissue architecture was performed by H&E staining to allow determination of histological score (Figure 3A) based on evaluation of tissue structural damage and infiltration of neutrophils through the mucosa (Figure S2A, B). Alcian blue staining of colonic tissue sections was performed to allow quantification of the loss of mucus‐containing goblet cells typically associated with DSS colitis (Figure 3B). Representative H&E and Alcian blue stained tissue sections are shown for healthy, DSS‐only, and WMP‐treated groups (Figure 3C). There was no difference in tissue damage or immune cell infiltration scores between DSS‐only and WMP pretreated groups, while both groups scored significantly higher compared to healthy controls (Figure S2A, B), resulting in no significant difference in total histological score (Figure 3A). Although not statistically significant, mucosa‐blood flux of FITC‐dextran appeared lower in mice pretreated with WMP compared to DSS‐only mice, returning to serum levels similar to healthy controls (Figure S2C). Calculation of goblet cell numbers indicated that the loss of goblet cells was less significant in the WMP‐treated group compared to DSS‐only mice (Figure 3B). To further investigate the effects of WMP treatment on intestinal barrier function, expression of genes encoding tight junction proteins occludin (Figure 3D) and claudin1 (Figure 3E), and the EGFR (Figure 3F) were determined in colonic tissue. Occludin expression was significantly increased in DSS mice compared to healthy control; however, it appeared significantly reduced in WMP‐treated mice compared to DSS only (Figure 3D). There was a significant increase in expression of claudin1 (Figure 3E) and EGFR (Figure 3F) in the WMP‐treated mice compared to healthy controls. No significant differences were observed between WMP‐treated and DSS‐only groups for CDH1, CLDN2, mucin (MUC)1, and zonula occludens (ZO)2 (Figure S3A–D).

### 3.5 *A. bisporus* WMP Improves Tissue Recovery From DSS‐Induced Colitis

Histological analysis of colonic tissue architecture was performed following recovery from DSS‐induced colitis by H&E staining to allow determination of histological score (Figure 3G) based on evaluation of tissue structural damage and infiltration of neutrophils through the mucosa (Figure S2D, E). Alcian blue staining of colonic tissue sections following the recovery period was performed to allow quantification of goblet cell numbers (Figure 3H). Representative H&E and Alcian blue stained tissue sections are shown for Healthy, DSS‐only, and WMP‐treated groups following recovery from DSS‐induced colitis (Figure 3J). Although not significant, there was a trend toward reduced tissue damage (Figure S2D) and immune cell infiltration (Figure S2E), in the distal colon in the WMP‐treated group compared to DSS‐only resulting in a similar nonsignificant reduction in total histology score (Figure 3G) potentially indicating enhanced tissue recovery. DSS‐only mice exhibited a significant increase in crypt length, which was not observed in WMP‐treated mice, supporting the suggestion that WMP administration promotes tissue recovery (Figure 3I). Goblet cell measurements appeared to have recovered in both DSS‐only and WMP‐treated groups at the end of the recovery period, with no significant differences observed (Figure 3H). RT‐qPCR analysis revealed a consistent increase in occludin transcription in the DSS‐only but not WMP‐treated group (Figure 3K). No significant differences in gene transcription were detected in claudin1 (Figure 3L) and EGFR (Figure 3M) or in other genes analyzed (Figure S3E–H).

### 3.6 *A. bisporus* WMP Pretreatment Reduced Proinflammatory Cytokines Proximal Tissue of DSS‐Induced Colitis

**FIGURE 4:**
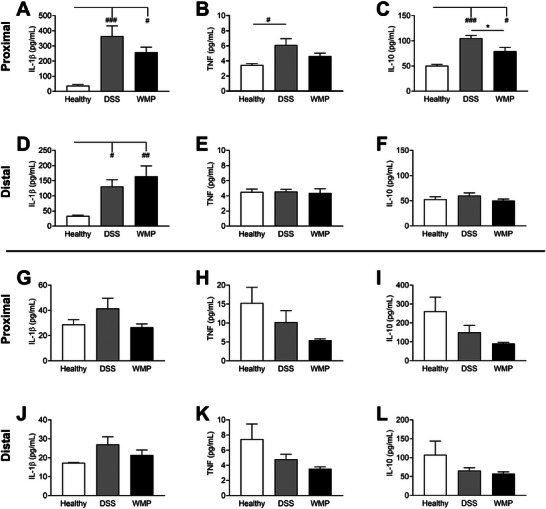
WMP has antiinflammatory effects in DSS‐induced colitis. Proximal and distal colon tissue from pretreatment (A–F) and recovery (G–L) trials were assayed by ELISA for cytokine levels of IL‐1β (A, D, G, J), TNF (B, E, H, K), and IL‐10 (C, F, I, L). Data are mean ± SEM of *n* = 5–6 mice per group. *p value < 0.05, **p value < 0.01, ***p value < 0.001 determined using one‐way ANOVA with Tukey post‐hoc multiple comparisons where appropriate. (# Relative to healthy control; * relative to DSS control). ANOVA, analysis of variance; DSS, dextran sulfate sodium; TNF, tumor necrosis factor; WMP, whole mushroom powder.

To further investigate the protective effects of *A. bisporus* WMP treatment on development of colitis, cytokine levels were quantified by ELISA in proximal and distal colon tissue (Figure 4A–F) and in serum (Figure S4A–C). IL‐1β levels in proximal colonic tissue were significantly increased in DSS‐only mice compared to healthy controls, with a less significant increase observed in WMP‐treated mice (Figure 4A). In distal colon however, IL‐1β levels were significantly increased in the WMP group compared to DSS‐only mice (Figure 4D). TNF levels were significantly increased in the proximal colon of DSS‐only but not WMP‐treated mice (Figure 4B) with no significant differences in the distal colon across all groups (Figure 4E). The amount of antiinflammatory IL‐10 was significantly increased in DSS‐only mice compared to healthy controls, and significantly reduced in the proximal colonic tissue of WMP mice compared to DSS‐only mice (Figure 4C). There was no difference in IL‐10 in distal colonic tissue across all groups (Figure 4F). However, serum IL‐10 was increased in both DSS‐only and WMP‐treated mice compared to the healthy controls (Figure S4C). Taken together, this suggests that WMP treatment has an antiinflammatory effect during colitis, primarily in the proximal colon.

### 3.7 *A. bisporus* WMP Treatment Does Not Affect Cytokines During Recovery From DSS‐Induced Colitis

The antiinflammatory potential of *A. bisporus* WMP was also investigated in proximal and distal colon tissue from mice in recovery (Figure 4G–L). Although no statistically significant differences were observed between WMP‐treated and DSS‐only mice for all cytokines analyzed, there was a subtle reduction in IL‐1β in the proximal and distal colon (Figure 4G, J) and for TNF in proximal tissue (Figure 4H) for the WMP group compared to DSS‐only. There were no differences observed in serum levels for all cytokines tested between healthy controls, DSS‐only, and WMP‐treated mice (Figure S4D–F).

### 3.8 *A. bisporus* WMP Pretreatment Impacts Oxidative Stress During DSS‐Induced Colitis

**FIGURE 5:**
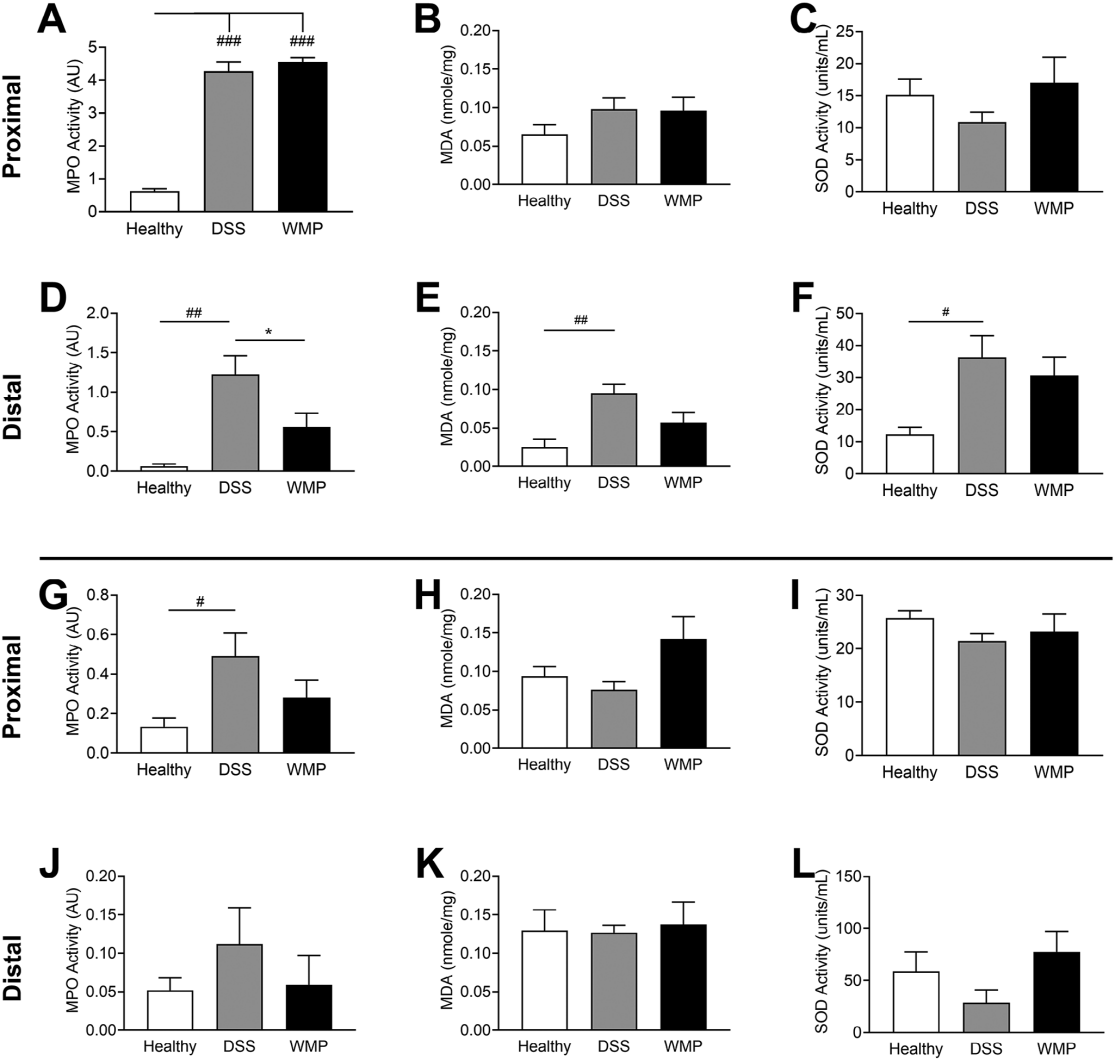
WMP has antioxidant effects in DSS‐induced colitis. Proximal and distal colon tissue from pretreatment (A–F) and recovery (G–L) trials were analyzed for levels of myeloperoxidase (MPO; A, D, G, J), malondialdehyde (MDA; B, E, H, K), and superoxide dismutase (SOD; C, F, I, L). Data are mean ± SEM of *n* = 5–6 mice per group. *p value < 0.05, **p value < 0.01, ***p value < 0.001 determined using one‐way ANOVA with Tukey post‐hoc multiple comparisons where appropriate. (# Relative to healthy control; * relative to DSS control). ANOVA, analysis of variance; DSS, dextran sulfate sodium; WMP, whole mushroom powder.

MPO activity, an indicator of neutrophil infiltration and oxidative stress, was assayed in proximal and distal colonic tissue. DSS administration increased MPO levels to similar levels in DSS‐only and WMP groups compared to healthy controls (Figure 5A); however, MPO levels were significantly lower in the WMP group compared to DSS‐only in the distal colon (Figure 5D). MDA, a marker of lipid peroxidation and oxidative stress, was increased to similar levels in proximal tissue of DSS‐only and WMP groups compared to healthy controls (Figure 5B), while in the distal colon there was an increase in MDA levels compared to healthy control in the DSS‐only mice but not WMP mice (Figure 5E). SOD activity, an antioxidant enzyme that helps break down reactive oxygen species (ROS), appeared increased in healthy controls and WMP‐treated proximal tissue compared to DSS‐only mice (Figure 5C). In contrast, SOD was significantly increased in distal colon of DSS‐only mice compared to healthy controls, with a slight reduction in WMP‐treated mice (Figure 5F).

### 3.9 *A. bisporus* WMP Treatment Impacts MPO Activity During Recovery From DSS‐Colitis

MPO levels were significantly increased in the proximal colon of the DSS group compared to healthy controls but were not significantly elevated in the WMP group, potentially indicating an improved recovery in WMP‐treated mice (Figure 5G). No significant differences were observed in MDA (Figure 5H) or SOD (Figure 5I) within proximal colonic tissue of all groups following the recovery period. In the distal colon, there was a trend for lower MPO levels in the WMP group compared to DSS‐only, reflective of MPO activity in the proximal colon; however, this was not statistically significant (Figure 5J). No significant differences in MDA levels (Figure 5K) or SOD levels (Figure 5L) were detected in distal colon across all groups. Taken together, these data suggest that WMP administration can modulate MPO activity to support recovery from colitis.

### 3.10 *A. bisporus* WMP Treatment Impacts Gut Bacterial Composition

**FIGURE 6:**
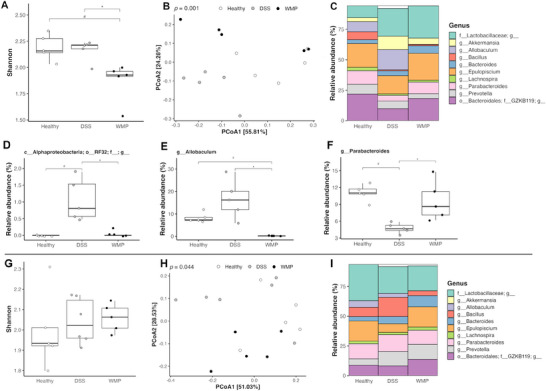
WMP affects bacterial composition of the gut before DSS‐induced colitis. Fecal samples from pretreatment (A–F) and recovery (G–I) trials were analyzed by 16S rRNA sequencing. In the pretreatment trial, alpha diversity is estimated by Shannon index (A) and beta diversity by PCoA analysis (B) at Day 9 (pre‐DSS). Relative abundance of the Top 10 genera at Day 9 are displayed as stacked bar chart (C). Differences between the WMP pretreatment compared to vehicle control at Day 9 were revealed in abundance levels of A*lphaproteobacteria* (D), *Allobaculum* (E), and *Parabacteroides* (F). In the recovery trial alpha diversity is estimated by Shannon index (G) and beta diversity by PCoA analysis (H) at Day 9 (trial end). Relative abundance of the Top 10 genera at Day 9 are displayed as stacked bar chart (I). Data represent *n* = 5–6 mice per group. *p value < 0.05 determined using PERMANOVA for PCoA plot and Wilcoxon signed‐rank test for differential abundance analysis. (# Relative to healthy control; * relative to DSS control). 16S rRNA, 16S ribosomal RNA; DSS, dextran sulfate sodium; PCoA, principal coordinate analysis; WMP, whole mushroom powder.

To determine any effect of WMP treatment on the gut microbiota and inflammation‐induced bacterial dysbiosis, we conducted 16S rRNA sequencing on fecal samples collected on Day 0, Day 9, and Day 16 in the pretreatment trial, with Day 9 (pre‐DSS), and Day 16 (post‐DSS) allowing analysis of WMP impact during both the pretreatment and DSS phases (Figure 6A–F). The Shannon index, as a measure of alpha diversity, was lower in the WMP group at Day 9 compared to both healthy control and vehicle control groups (Figure 6A), and beta diversity illustrated by PCoA plot indicates a significant difference in microbiome structure across all groups (Figure 6B). The relative abundance of the Top 10 bacterial species was identified at Day 9 (Figure 6C). Taxonomic analysis at the genus level revealed a significant decrease in the abundance of *A*lphaproteobacteria and Allobaculum species, and an increase in Parabacteroides species in the WMP‐treated group compared to vehicle controls (Figure 6D–F). At this pre‐DSS timepoint, it was noted that the vehicle control group showed a significantly increased relative abundance of *Alphaproteobacteria* and a decrease in *Parabacteroides* compared to the healthy control receiving sham gavage. While unexpected, PBS gavage may subtly influence the microbiome by altering mucosal hydration, gut motility, or pH, potentially creating minor shifts in the microbial environment to favor certain taxa. Therefore, we limit comments to comparisons between the vehicle and WMP groups to focus on treatment‐specific effects. All species indicating significant differences in relative abundance by differential abundance analysis at this timepoint are shown in Figure S5. At Day 16 no difference was observed in alpha diversity on the Shannon index (Figure S5C), although the PCoA plot indicates a significant difference across groups at this timepoint; this is due to the effects of DSS‐induced colitis compared to healthy controls (Figure S5B). There was no difference in the relative abundance of bacterial species at Day 16 (Figure S5C).

### 3.11 *A. bisporus* WMP Treatment Does Not Affect Bacterial Composition of the Gut During Recovery From DSS‐Induced Colitis

To determine any effect of WMP treatment on the gut microbiota during the recovery period following DSS colitis, we conducted 16S rRNA sequencing on fecal samples from the recovery trial (Figure 6G–I). On the final day of the trial, we observed no significant difference in alpha diversity as measured by the Shannon index (Figure 6G). Although beta diversity illustrated by PCoA plot indicates differential gene expression across groups (Figure 6H). This difference in microbiome structure is likely due to the effects of DSS‐induced colitis compared to healthy controls. The relative abundance of the Top 10 bacterial species was identified (Figure 6I). However, differential abundance analysis did not reveal any significant differences in bacterial composition at this timepoint.

## 4 Discussion

IBD is a chronic inflammatory condition of the digestive tract. Clinically, IBD is characterized by symptoms such as diarrhea, abdominal pain, rectal bleeding, weight loss, and fatigue. A key pathophysiological feature of IBD is increased intestinal permeability, often referred to as a “leaky gut,” wherein the integrity of tight junctions within the intestinal epithelium is compromised, permitting the translocation of bacteria and antigens across the intestinal barrier and into the bloodstream, contributing to the pathogenesis of the disease [44].

Recent research has highlighted the potential therapeutic benefits of polysaccharide extracts and products derived from various mushroom species in experimental models of IBD [19, 45–47]. Notably, the therapeutic effects of extracts from white button mushroom *A. bisporus* were observed in DSS‐induced colitis. Liu et al. (2024) [28] reported that polysaccharide extracts derived from *A. bisporus* provide protection from weight loss, colon atrophy, and histological damage, an increase in tight junction proteins, restoration of gut–barrier function, and inhibition of inflammation that were associated with microbiota changes. The current study sought to investigate the beneficial “entourage effect” of WMP derived from *A. bisporus*, and it is potential to both protect against and promote healing in gastrointestinal inflammation. Indeed, our prior preliminary work indicated that *A. bisporus* WMP pretreatment could reduce IL‐1β and IL‐6, and MPO activity in the proximal colon of a murine DSS‐induced colitis model [23].

We previously established an in vitro digestion procedure for WMP and confirmed that, following simulated digestion, IVD‐WMP retains its antiinflammatory properties in HEK dectin1 and HEK Toll‐like‐receptor‐4 cell lines. Additionally, we demonstrated that intraperitoneal injection of IVD‐WMP is antiinflammatory in a mouse model of zymosan‐induced peritonitis [23]. We later showed that IVD‐WMP maintains its β‐glucan content compared to WMP and retains the capacity to induce trained immunity, enhancing inflammatory responses in human monocyte‐derived macrophages [24]. To simulate the physiological conditions of WMP more accurately in the colon, we carried out in vitro digestion of WMP and used this IVD‐WMP in our in vitro assays on human epithelial cell lines. Our investigation of A. bisporus WMP's direct effects on epithelial barrier integrity revealed reduced permeability to FITC‐dextran in both Caco‐2 and HT‐29‐MTX monolayers. These findings suggest that enhanced gastrointestinal barrier protection and repair may contribute to the observed decrease in permeability. Previous studies have indicated that A. bisporus extracts exhibit antiinflammatory and antioxidant effects in Caco‐2 cells stimulated with LPS and TNF, reducing COX‐2 and PF_2_AR expression, decreasing levels of IL‐6 and increasing NRF‐2 [25]. Disruption of intestinal tight junction complexes and mucus layer have been observed in IBD patients and contribute to impaired barrier function and “leaky gut” [48]. Our in vitro findings align with the results of our DSS‐induced colitis trials, where WMP decreased intestinal permeability, suggesting that A. bisporus WMP plays a beneficial role in protecting and repairing gastrointestinal barrier integrity. However, it is important to note that our in vitro digestion protocol does not account for the effects of gastrointestinal microbiota on WMP digestion. Although it appears the IVD‐WMP product can influence immune responses and barrier function in our in vitro work on cell lines, given the microbiota‐related effects observed in our study, it is plausible that the full potential of WMP may not be fully realized in the absence of microbial interactions.

The current investigation employed a DSS‐induced colitis model in mice to evaluate the effects of A. bisporus WMP in both a preventative pretreatment context and a therapeutic recovery context. Due to ethical concerns, best practice guidelines, and project license limitations on repeated oral gavage in mice, it was not possible to administer WMP before, during, and following colitis in the same cohort of mice. However, we would hypothesize that this dosing schedule could contribute to further amelioration of the beneficial effects described, as WMP pretreatment seems to moderately decrease the severity of inflammation and, following inflammation, to speed repair of the tissue. Oral gavage was chosen as the most efficient way of ensuring that dosing of WMP was consistent for each mouse during the trial, particularly during and after the DSS period when food intake tends to be lower. However, this procedure can be stressful; therefore, long‐term effects of dietary WMP, both generally and pre‐, during, and postinflammation could be studied in the future by incorporation into standard mouse chow.

Although the effects were not significant, pretreatment with WMP consistently scored lower in stool and blood indices during the acute DSS colitis period and provided significant protection against colon shortening at the day of sacrifice. Histologically, no benefits of WMP pretreatment were observed in distal colon tissue in terms of tissue damage or cell infiltration; however, protection against goblet cell loss was observed. Additionally, there was a nonsignificant trend for intestinal permeability, as measured by FITC‐dextran, to be protected by WMP pretreatment. When administered during the recovery phase following DSS‐induced colitis, WMP significantly lowered stool and DAI scores, and protected against colon shortening. Tissue histology showed improved crypt length measurements and a subtle, nonsignificant improvement in tissue damage and neutrophil infiltration scores in the WMP group, which may be suggestive of a boost to tissue repair.

Intestinal epithelial barrier integrity is maintained via tight junction complexes, which are crucial for regulating transport across the epithelium. Disruption of tight junction proteins by dysregulated protein expression has been implicated in the pathogenesis of IBD [49]. Mice pretreated with WMP exhibited a trend for increased transcription of genes associated with barrier integrity and epithelial protection compared to controls. Notably, there was a significant upregulation of the tight junction protein claudin1 and the EGFR in WMP pretreated mice compared to healthy control. The fact that DSS groups have increased expression of these genes compared to healthy mice is surprising but may signify that repair has already begun at this timepoint, which was 2 days post‐DSS cessation. We found the significant increase in occludin expression in DSS mice surprising, as occludin loss has been associated in vitro and in vivo with increased epithelial tight junction permeability induced by inflammatory stimuli. This may again be due to the timepoint at which mice were culled. This timepoint was chosen as it typically demonstrates the peak of overall disease activity scores, though it is likely that recovery processes have begun, considering the decreased blood scores we see at this timepoint. Interestingly, recent evidence demonstrates that occludin KO mice are resistant to DSS‐induced colitis due to an adaptive mechanism whereby loss of occludin transcription is associated to suppression of the caspase3 apoptosis pathway [50].

Inflammatory mediators and ROS play a critical role in the disruption of tight junction proteins and the subsequent increase in gut permeability that contributes to the progression of ulcerative colitis [51]. Therefore, we investigated the effects of WMP on cytokine levels and oxidative stress markers in our murine colitis models. In the pretreatment trial, IL‐10 levels were significantly lower in the proximal colon of WMP pretreated mice compared to DSS mice, although both groups showed increased levels compared to healthy controls. This finding is unexpected given that IL‐10 is typically reduced in DSS; when given as a treatment, IL‐10 reduces DSS severity [52], and DSS colitis is exacerbated in IL‐10‐deficient mice [53]. Indeed, a recent paper utilizing a strain of *E. coli* known to increase IL‐10 macrophages demonstrated an amelioration of DSS colitis in mice [54].

We previously reported a decrease in IL‐10 in the proximal colon in DSS with some protection against this decrease for WMP [23]. We also observed that WMP attenuated the DSS‐induced increases in IL‐1β and IL‐6. In the current study, while we observed attenuation of IL‐1β and TNF‐α, the previously noted protection against IL‐6 was not replicated (not shown). In the distal colon, IL‐1β levels were more significantly increased in the WMP group than DSS. However, our previous study used a lower dose of WMP given daily rather than every 2nd day, and mice were culled at DSS cessation rather than 2 days later as reported here. In the literature, most DSS studies of medicinal mushroom polysaccharide extracts demonstrate a reduction in proinflammatory mediators and a boost to antiinflammatory cytokines in the colon [46, 47] and typically also cull mice immediately on the final day of DSS. Examining cytokine levels in both the proximal and distal colon in the recovery trial, there were no statistically significant differences. However in the proximal colon, we see a similar nonsignificant trend for reduced IL‐1β in the proximal colon.

As expected, oxidative stress and lipid peroxidation, indicated by increased levels of MPO and MDA, were elevated following DSS‐induced colitis compared to controls. WMP pretreatment could significantly mitigate MPO increases and similarly showed a trend suggesting protective effects on MDA levels, in the distal colon compared to DSS. SOD, an antioxidant enzyme that is active in response to ROS, was increased in the distal colon of both groups likely in response to DSS‐induced ROS production. In the recovery trial, MPO levels in the proximal colon approached those of healthy controls for the WMP group, while the DSS group was still significantly elevated. In the distal colon, we see a similar, though nonsignificant, trend again indicating a faster recovery. No significant differences in MDA or SOD activity were observed across any groups in recovery. These findings suggest that WMP may support intestinal barrier integrity in DSS‐induced colitis by modulating inflammation and oxidative stress. Previous in vitro studies have demonstrated significant DPPH scavenging ability of methanolic [55] and ethanolic [56] extracts from *A. bisporus*. The study examining ethanolic extracts also reported strong reducing power, as well as potent superoxide and hydroxyl radical scavenging activities, along with moderate hydrogen peroxide scavenging capacity. Furthermore, in vivo administration of *A. bisporus* ethanolic extract significantly enhanced antioxidant activity in murine serum, liver, and heart tissues [56]. Recent reports have demonstrated similar results regarding the health impacts of polysaccharide compounds from various mushroom species, including their roles in immunomodulation and oxidative stress regulation [57, 58]. Taken together, our observations and previous literature demonstrate that mushroom extracts are a source of natural antioxidants, with the consumption of mushrooms and mushroom‐derived extracts potentially providing a degree of protection against oxidative damages.

To evaluate the potential modulatory effects of WMP on gut microbiota, we performed 16S rRNA sequencing on fecal samples collected from mice across both experimental trials. In the pretreatment trial, we observed a lower Shannon index, indicative of reduced alpha diversity, in the WMP group compared to the DSS group on Day 9. Given that this finding was also observed on Day 0 (data not shown), we believe it to be a cage effect, unrelated to WMP treatment or DSS, as mice were housed according to treatment group. Beta diversity metrics indicated a significant overall difference in microbiome composition across groups at Days 9 and 16. A more detailed taxonomic analysis at the genus level revealed differences in the WMP pretreated group compared to vehicle control, indicating that WMP may selectively influence specific bacterial taxa. These changes were noted at Day 9 only, before DSS administration. There was a significant reduction in the relative abundance of *Alphaproteobacteria* and the genus *Allobaculum*, while *Parabacteroides* were increased in the WMP group compared to vehicle control. *Alphaproteobacteria* are a class of bacteria in the phylum Pseudomonadota. The relative abundance of the RF32 order has previously been positively correlated with increased histopathology and colonic inflammation in mice with adoptive transfer colitis [59]. The *Allobaculum* genus, belonging to the *Erysipelotrichaceae* family, is an underexplored member of the intestinal microbiota. However, they have been implicated in inflammatory processes. A positive correlation between *Allobaculum* abundance and levels of ileal RORγT and IL‐17 has been reported in mice [60], while Miyauchi et al. (2020) [61] linked a specific *Allobaculum* strain (OTU002) to increased susceptibility to experimental autoimmune encephalitis. This strain's ability to adhere to small intestinal epithelial cells was found to induce the expansion of inflammatory T helper 17 cells. Notably, *A. mucolyticum* was initially isolated from an ulcerative colitis patient in a study where bacteria selected on the basis of high levels of coating with IgA conferred dramatic susceptibility to DSS‐induced colitis in germ‐free mice [62]. More recently *A. mucolyticum* was shown to be an effective degrader of MUC, which suggests that it could contribute to IBD pathogenesis [63]. This speaks to our finding in the pretreatment trial of lesser goblet cell loss in the WMP pretreatment group. These findings suggest that *Allobaculum* species may play a role in the development of intestinal inflammation. *Parabacteroides* is a relatively new genus, established following taxonomic reclassification in 2006, with Parabacteroides distasonis serving as the reference strain. P. distasonis has demonstrated both beneficial and pathogenic effects in murine colitis models, which appear to be context‐dependent [64]. Studies have shown that administration of P. distasonis can alleviate symptoms in two experimental colitis models [65, 66]. However, its abundance has also been observed to increase in acute and chronic DSS‐induced colitis models [38]. Additionally, levels of P. distasonis have been found to inversely correlate with remission duration in patients with ulcerative colitis [67]. In the recovery trial, although beta diversity analysis indicated a significant difference in microbial composition across the groups, this is likely due to the effects of colitis. Taxonomic analysis did not identify differences in the relative abundance of specific bacterial genera at any timepoint.

## 5 Conclusion

In conclusion, this study demonstrates that WMP from *A. bisporus* can ameliorate colitis symptoms and aid recovery through mechanisms associated with gastrointestinal barrier support, immune modulation, oxidative stress regulation, and specific modulation of gut microbiota composition. This is the first comprehensive investigation of the effects of *A. bisporus* WMP on immunomodulation, oxidative stress regulation, and gut microbiota changes when given as preventative or curative treatment in a DSS‐induced colitis mouse model. These findings contribute to our understanding of the protective effects of *A. bisporus* mushroom powder in colitis and suggest that whole *A. bisporus* or its extracts could be utilized as a new natural antioxidant and antiinflammatory nutraceutical or dietary component in food and therapeutics.

## Conflicts of Interest

Supriya Yadav and Jude Wilson are employees of MBio, part of Monaghan Mushrooms Group who supplied the powdered mushroom used in this study. The remaining authors declare that the research was conducted in the absence of any commercial or financial relationships that could be construed as a potential conflict of interest.

## Supporting information




Supporting file 1: mnfr70133‐sup‐0001‐SuppMat.docx

## Data Availability

The data that support the findings of this study are available from the authors upon reasonable request.
