# Supplementary material for: Protective Properties of the White Button Mushroom, Agaricus bisporus, in a Mouse Model of Colitis
Source: Mol Nutr Food Res. 2025 Jun 12;69(19):e70133. doi: 10.1002/mnfr.70133 (PMC12490186; doi:10.1002/mnfr.70133)
Supplement: Supplementary file 1 — Supporting file 1: mnfr70133‐sup‐0001‐SuppMat.docx [file MNFR-69-e70133-s001.docx]

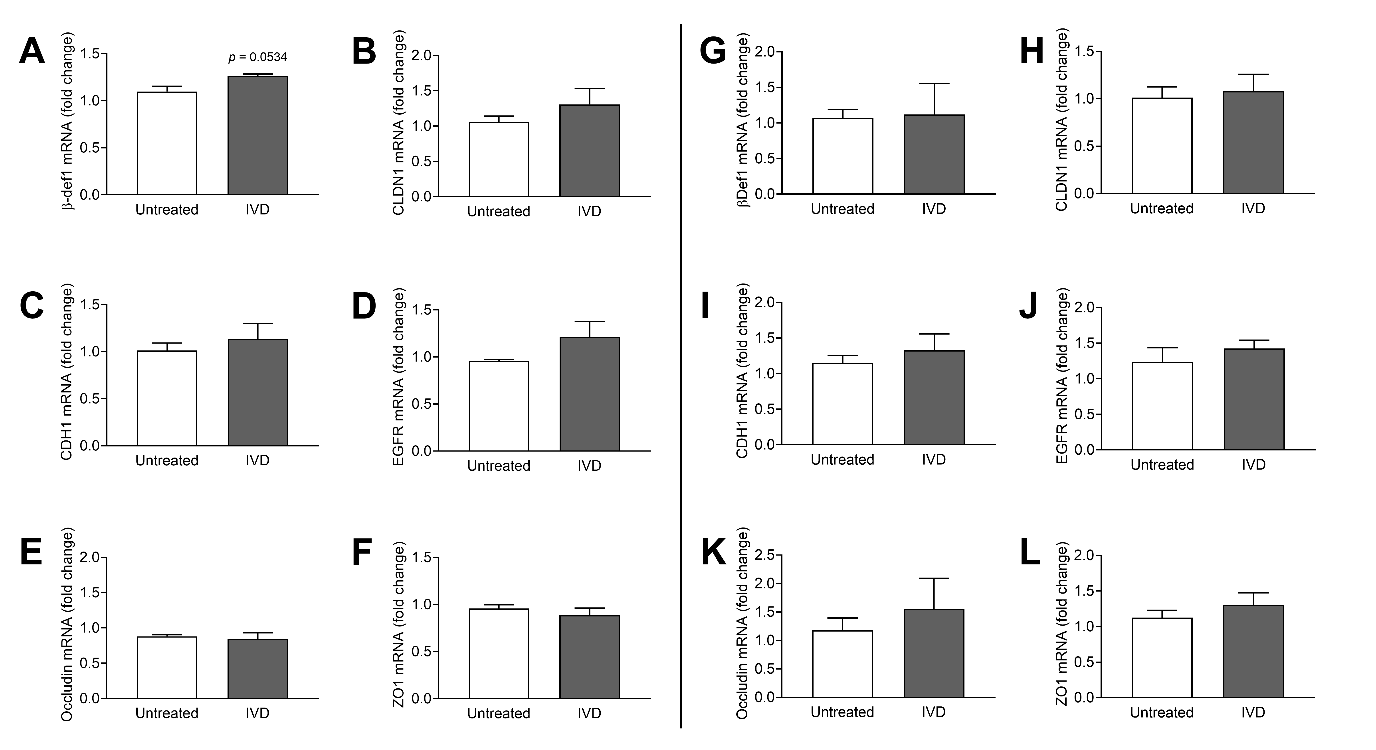


**Supplementary Figure 1 – Impact of IVD-WMP on barrier-related gene expression in Caco-2 or HT-29-MTX cells**

**Caco-2 (A-F) and HT-29-MTX (G-L) cell lines were grown on transwell plates and pre-treated for 24h with *A. bisporus* IVD-WMP (1mg/mL). Fold change in expression of genes related to barrier integrity were measured: β-defensin1 (β-def1; A,G), claudin1 (CLDN1; B,H), e-cadherin (CDH1; C,I), EGFR (D,J), occludin (E,K), and zonula occludens1 (ZO1; F,L). Data are mean ± S.E.M. of *n* = 3 independent experiments (unpaired *t*-test).**


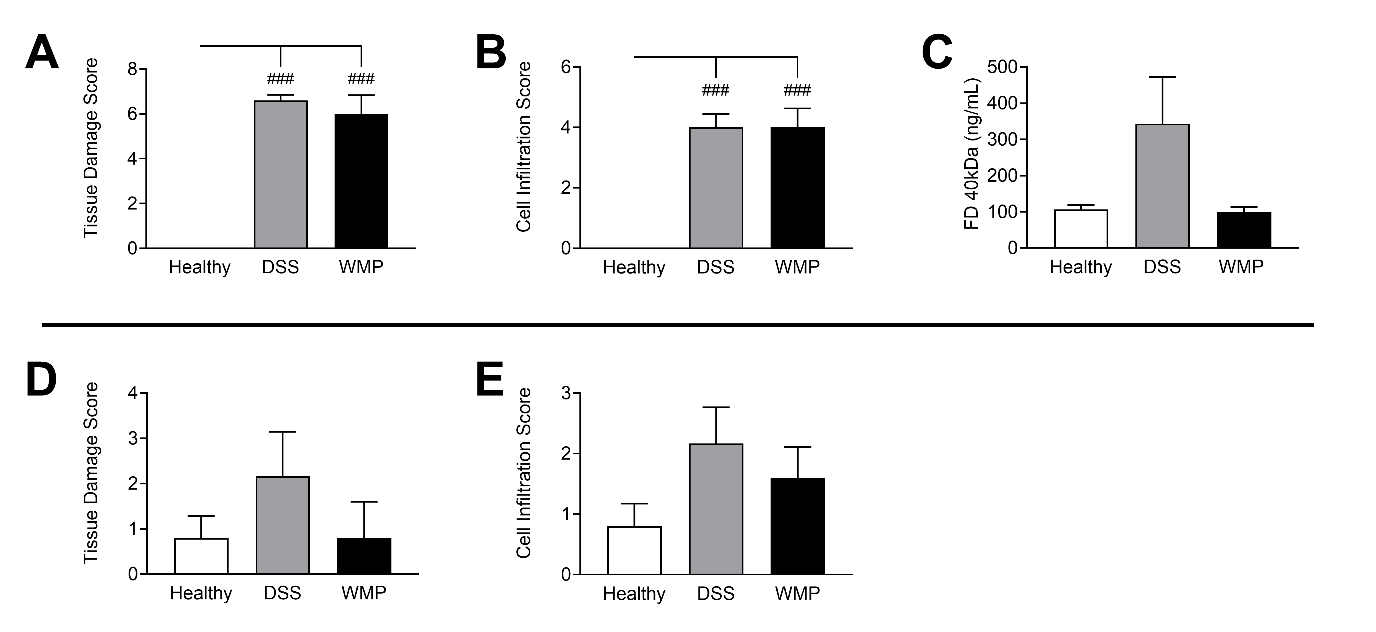


**Supplementary Figure 2 – Impact of WMP on tissue damage, cell infiltration and intestinal permeability in DSS-induced colitis mice**

**Distal colon tissue from pre-treatment (A, B) and recovery (D, E) trials were analysed by H&E for tissue damage (A, D) and neutrophil infiltration (B, E). Pre-treatment mice were assessed for mucosa-blood flux of FITC-dextran (40kDa) which was administered orally 4h prior to cull and subsequently measured in serum (C). Data are mean ± S.E.M. of *n* = 5-6 mice per group. ****P* value < 0.001 determined using one-way ANOVA with Tukey post-hoc multiple comparisons where appropriate (# relative to healthy control; * relative to DSS control).**


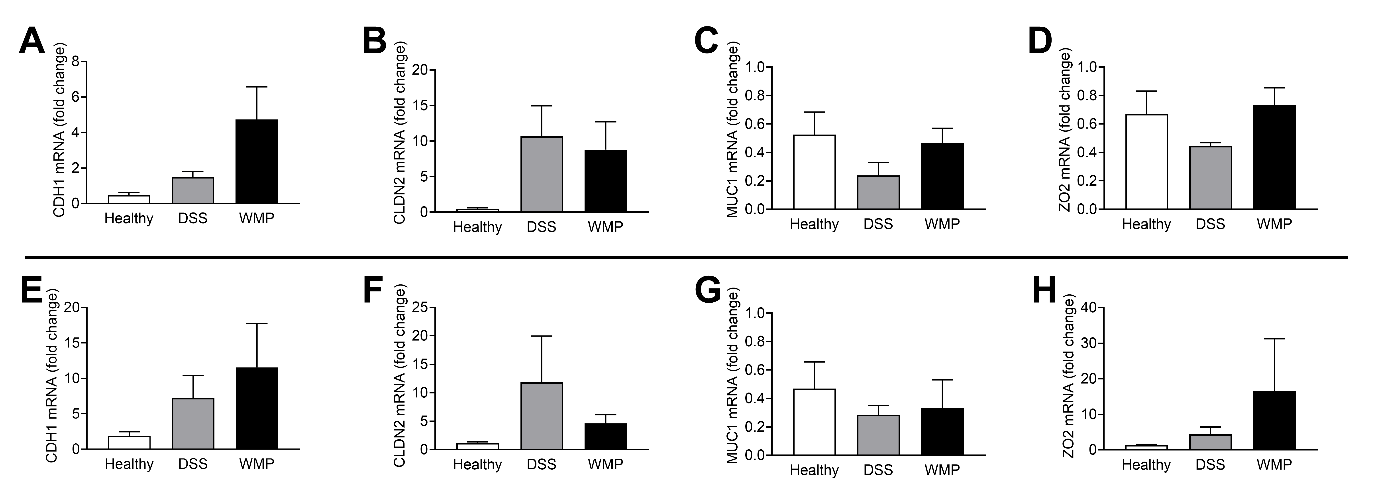


**Supplementary Figure 3 – Impact of WMP on barrier-related gene expression in DSS-induced colitis mice**

**RNA was extracted from distal colon tissue from pre-treatment (A-D) and recovery (E-H) trials and fold change in expression of genes related to barrier integrity were measured: e-cadherin (CDH1; A,E), claudin2 (CLDN2; B,F), mucin1 (MUC1; C,G), zonula occludens2 (ZO2; D,H). Data are mean ± S.E.M. of *n* = 3-5 mice per group (one-way ANOVA with Tukey post-hoc multiple comparisons where appropriate).**


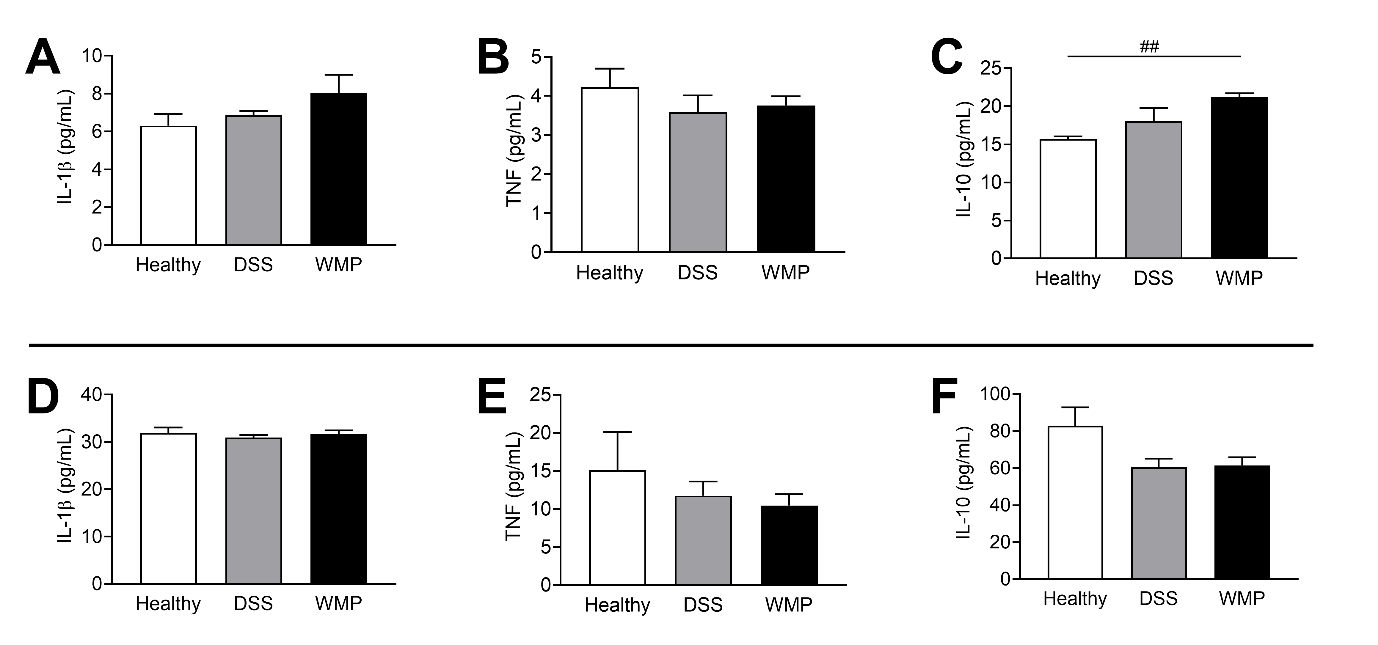


**Supplementary Figure 4 – WMP increases IL-10 in serum of DSS-induced colitis mice**

**Serum from pre-treatment (top) and recovery (bottom) trials were assayed by ELISA for cytokine levels of IL-1β (A, D), TNF (B, E), and IL-10 (C, F). Data are mean ± S.E.M. of *n* = 5-6 mice per group. ***P* value < 0.01 determined using one-way ANOVA with Tukey post-hoc multiple comparisons where appropriate. (# relative to healthy control; * relative to DSS control).**


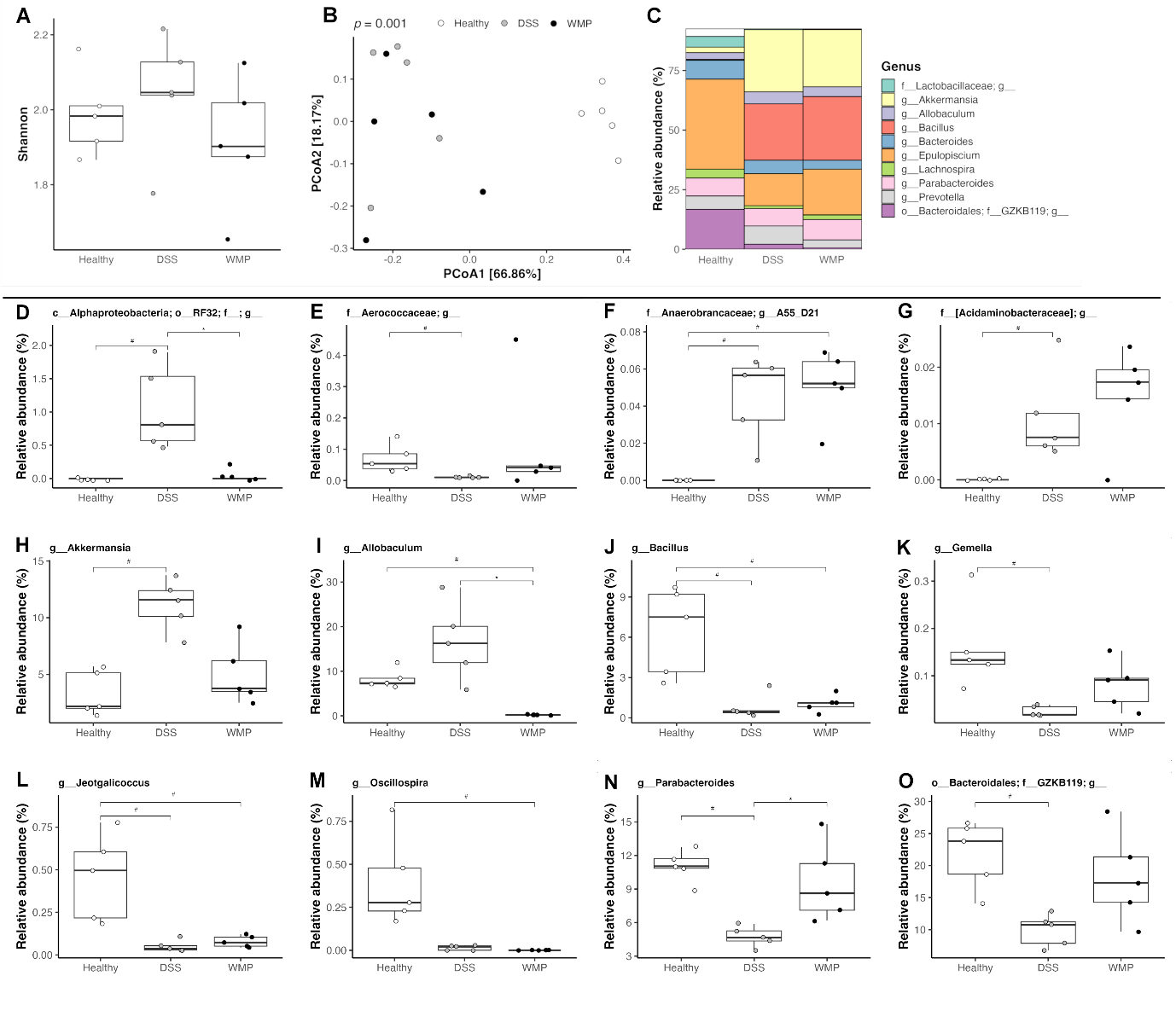


**Supplementary Figure 5 – WMP does not affect bacterial composition of the gut following DSS-induced colitis**

**Fecal samples from day 16 (A-C) and day 9 (D-O) of the pre-treatment trial were analysed by 16S rRNA sequencing. Alpha diversity is estimated by shannon index (A) and beta diversity by PCoA analysis (B) at day 16 (post-DSS). Relative abundance of the top 10 genera at day 16 are displayed as a stacked bar chart (C). Differences between groups at day 9 were revealed in abundance levels: A*lphaproteobacteria* (D), *Aerococcaceae* (E), *Anaerobrancaceae* (F), *Acidaminobacteraceae* (G), *Akkermansia* (H), *Allobaculum* (I), *Bacillus* (J), *Gemella* (K), *Jeotgalicoccus* (L), *Oscillospira* (M), *Parabacteroides* (N) and *Bacteroidales* (O). Data represent *n*= 5 mice per group. **P*value < 0.05, ***P*value < 0.001 determined using PERMANOVA for PCoA plot and Wilcoxon Signed-Rank Test for differential abundance analysis.  (# relative to healthy control; * relative to DSS control).**
